# Split-based computation of majority-rule supertrees

**DOI:** 10.1186/1471-2148-11-205

**Published:** 2011-07-13

**Authors:** Anne Kupczok

**Affiliations:** 1 Center for Integrative Bioinformatics Vienna, Max F. Perutz Laboratories, University of Vienna, Medical University of Vienna, University of Veterinary Medicine Vienna, Dr. Bohr-Gasse 9, A-1030 Vienna, Austria; 2 Institute of Science and Technology, Austria, Am Campus 1, 3400 Klosterneuburg, Austria

## Abstract

**Background:**

Supertree methods combine overlapping input trees into a larger supertree. Here, I consider split-based supertree methods that first extract the split information of the input trees and subsequently combine this split information into a phylogeny. Well known split-based supertree methods are matrix representation with parsimony and matrix representation with compatibility. Combining input trees on the same taxon set, as in the consensus setting, is a well-studied task and it is thus desirable to generalize consensus methods to supertree methods.

**Results:**

Here, three variants of majority-rule (MR) supertrees that generalize majority-rule consensus trees are investigated. I provide simple formulas for computing the respective score for bifurcating input- and supertrees. These score computations, together with a heuristic tree search minmizing the scores, were implemented in the python program PluMiST (Plus- and Minus SuperTrees) available from http://www.cibiv.at/software/plumist. The different MR methods were tested by simulation and on real data sets. The search heuristic was successful in combining compatible input trees. When combining incompatible input trees, especially one variant, MR(-) supertrees, performed well.

**Conclusions:**

The presented framework allows for an efficient score computation of three majority-rule supertree variants and input trees. I combined the score computation with a heuristic search over the supertree space. The implementation was tested by simulation and on real data sets and showed promising results. Especially the MR(-) variant seems to be a reasonable score for supertree reconstruction. Generalizing these computations to multifurcating trees is an open problem, which may be tackled using this framework.

## Background

Supertree methods amalgamate trees containing information from different, but overlapping, relationships into a larger supertree (e.g., [1]). The input trees need not have the same taxon sets, but the supertree contains all of the taxa present in at least one of the input trees. With this property, supertrees are applied to combine information present in different gene trees to infer relationships about larger sets of taxa (e.g., [2-6]).

Supertree methods can be distinguished by the elementary relationships they extract from the gene trees. These relationships can be splits (e.g., [7-9]), rooted triplets (e.g., [10-12]) or quartets (e.g., [13,14]). Here, I focus on split-based supertree methods. A *split *is a bipartition of the taxa and a split of a tree corresponds to an edge in the tree that divides these two sets. Splits are *compatible *if they can occur together in a tree. Otherwise they are *incompatible*. A *subsplit *of a supertree split is generated by deleting some taxa from the taxon set. Thus an input tree split may be subsplit of a supertree split. The definition of compatibility can also be applied to splits on overlapping taxon sets, then the splits are first reduced to the common taxa and subsequently tested for compatibility.

It is natural for split-based supertree methods to first extract the splits from the input trees and code them into a *matrix representation *(see e.g., the splits from  in Table 1). The most widely applied supertree method is matrix representation with parsimony (MRP, [7,8]). In this approach, the matrix representation is interpreted as a binary alignment and the supertree is the most parsimonious tree given the alignment. Matrix representation with compatibility (MRC, [9,15]) searches for the supertree maximizing the number of input splits that are a subsplit of a supertree split. MRC can also be understood as the compatibility method [16] applied to the binary alignment.

**Table 1:** Matrix representation and relationship matrix

Matrix representation							
							
		
	***s***_**1**_	***s***_**2**_	***s***_**3**_	***s***_**4**_	***s***_**5**_	***g***_**1**_	***g***_**2**_
A	1	1	1	0	0	-	-
B	1	1	1	0	0	-	-
C	0	1	1	0	0	0	0
D	0	0	1	0	0	1	0
E	0	0	0	0	0	-	-
F	0	0	0	1	0	1	0
G	0	0	0	1	1	0	1
H	0	0	0	1	1	0	1

**Relationship matrix**							
	***s***_**1**_	***s***_**2**_	***s***_**3**_	***s***_**4**_	***s***_**5**_	***c***_***i***_	

*g*_1_	c	c	i	i	c	1	
*g*_2_	c	c	c	c	s	0	

*b_j_*	0	0	1	1	0		

**Figure 1:**
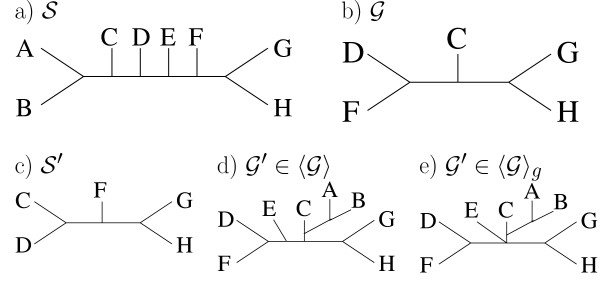
**Example input- and supertree**. Example supertree (a) and input tree (b) for distance computations. Both trees are identified by its split sets:  = {*AB*|*CDEFGH, ABC*|*DEFGH, ABCD*|*EFGH, FGH*|*ABCDE, GH*|*ABCDEF*} and  = {*DF*|*CGH, GH*|*CDF*} The supertree pruned to the taxa in  is shown in (c). Thus *d^- ^*= 2. (d) shows a tree that results in an optimal distance of *d*^+ ^= 4 and (e) shows a tree that results in an optimal distance of .

Coding of the example trees in Figure 1. Only inner splits are shown.

The task of summarizing trees on the same taxon set, the so called *consensus setting*, is well studied (e.g., [17,18]). Supertree methods can be understood as *generalizations *of consensus methods, that is, when applying a supertree algorithm in the consensus setting, the result should then be equivalent to the consensus. One popular consensus method is the *majority-rule *(MR) consensus, which produces a consensus tree that contains all splits present in at least half of the input trees. The MR consensus tree is a median tree under the Robinson-Foulds distance, in that, it is the tree with the smallest sum of the Robinson-Foulds distances to the input trees [19]. The *Robinson-Foulds *(RF) distance of two trees is the number of splits occurring in each of the trees, but are not found in the other [20].

Study of the consensus setting may also lead to important insights for supertree methods. Obviously, if a supertree method does not fulfill a property in the consensus setting, the property does not hold in general. For example, Wilkinson et al. [21] studied Pareto properties. They show that most supertree methods, including MRC and MRP, are Pareto on splits, i.e. the supertree contains a split if it is contained in all input trees. Methods are not co-Pareto on splits, if the supertree contains splits not supported by any input tree. E.g. MRC is co-Pareto on splits, but MRP is not. Some supertree methods show a bias in tree shape [22]. MRP shows a bias towards unbalanced shapes which is caused by the asymmetry of the underlying distance [23].

MRC and MRP can also be seen as median methods based on an *asymmetric *distance. The underlying distances are asymmetric since they only evaluate the fit of the input trees on the supertree and not vice versa. MRC is a median tree under the asymmetric RF distance, that is, the number of splits that are in the input tree but not in the pruned supertree. Thus, it generalizes the asymmetric median consensus [24,22]. Analogously, MRP can be interpreted as a median method based on the asymmetric parsimony distance.

Due to the asymmetric distances, MRC and MRP may favor relationships contradicting a majority of the input trees [25]. Cotton and Wilkinson [26] define majority-rule supertree methods as supertree methods generalizing the MR consensus. Different variants of MR supertrees exist and have been investigated. The main division among variants is between MR(-) and MR(+). The first evaluates distances between the pruned supertree and each input tree, while the second evaluates distances between the supertree and extended input trees. Here, *extension *refers to a method that adds missing taxa onto the input trees. Since this extension can be defined in multiple ways, multiple variants of MR(+) supertrees exist [26,27]. MR(+)s supertrees, a variant of MR(+) supertrees, can be solved exactly with an integer linear programming formulation [28]. MR(-) supertrees are closely related to RF supertrees [29] since both evaluate the RF distances between the pruned supertree and the input trees. Conceptually, there is a large difference between RF supertrees and MR supertrees. The aim of the first is to find at least one bifurcating tree of optimal score [29]. The approach of the latter, in contrast, is to find *all *trees of optimal score and to summarize them using the strict consensus method into a potentially multifurcating supertree. This also allows for labelling the MR supertree with values of support in the gene trees. By definition, finding *all *trees of optimal score also includes searching over multifurcating trees. Here a first attempt to solve the problem is performed that only searches for bifurcating trees of optimal score. From now on, I will also call these bifurcating trees of optimal score *supertrees*, since they contain all taxa from the input trees. They should not be confused with the MR supertrees that are obtained after the consensus step and can thus be multifurcating (see next section).

To date, there is only one study comparing the properties of different MR supertree variants [27] and I am not aware of any study on the performance of different MR supertree variants. The aim of this paper is to suggest a general framework for the distance computations underlying the MR supertree methods, to present an implementation evaluating different distance variants, and to compare these by simulation.

## Results and Discussion

### Algorithm

#### Score computation

I present the computation of majority-rule (MR) supertrees based on *bifurcating *and *unrooted *input- and supertrees. These trees contain only nodes with either one adjacent edge (the *terminal nodes *labeled with a taxon and the adjacent edge is a *terminal edge*) or with three adjacent edges (the *inner *nodes; non-terminal edges are also called *inner edges*). If an inner node of a tree has more than three adjacent edges, the tree is *multifurcating*. Note that bifurcating trees of *n *taxa have *n *- 3 inner edges and, in comparison, multifurcating trees of *n *taxa have fewer edges.

The Robinson-Foulds (RF) distance is only defined for trees labeled with the same taxa. There are two main ways to compute the RF distance between a supertree and an input tree when the input tree may contain only a subset of the taxa [26]:

1. Prune the supertree to the set of taxa in the input tree and compare the resulting tree to the input tree. This distance is called *d ^-^*.

2. Graft the remaining taxa in all possible ways onto the input tree, compute all distances, and take the minimal distance. There are different variants of grafting taxa onto input trees [27]. Here, two methods are investigated: (1) *d*^+ ^extends an input tree to all bifurcating trees by placing additional taxa onto edges only and by resolving multifurcations in the input trees; (2)  extends an input tree to bifurcating and multifurcating trees by placing taxa onto edges or nodes, but does not resolve multifurcations present in the input tree.

Example distance computations are shown in Figure 1.

The algorithm to compute the distances proceeds as follows. The matrix representation is a binary coding of the splits in the input- and supertree (Table 1). Next, a *relationship matrix *is computed from this matrix representation. For each (input split, supertree split) pair, the relationship matrix has three possible entries: subsplit; compatible but no subsplit; or incompatible. The relationship matrix of the example trees is given in Table 1. From this matrix, it is easy to see, whether the *i*-th input split is incompatible to at least one supertree split, then a binary variable *c_i _*is set to 1. Analogously, a binary variable *b_j _*is set to 1, if the *j*-th supertree split is incompatible to any input split. For a bifurcating input tree  and supertree , with  and *n *taxa, respectively, the distance computations can then be simplified as follows (see methods section):

The sum over the distances of all input trees  is called the *score *of a supertree with the respective supertree method, i.e., the score of  with MR(-) is , with MR(+) it is , and with MR(+)g it is . Note that the score of MR(-) also applies to multifurcating input trees (see methods section).

Here, *d*^+ ^and  differ only by the way the taxa are placed because of the restriction to bifurcating input trees. When computing *d*^+^, taxa can only be placed onto edges, and when computing  taxa can be placed onto edges or nodes. Note that MR(+) does not generalize majority-rule consensus but rather another consensus method called majority-rule(+) consensus [28,18]. Here, I deal only with bifurcating input trees. It is easy to see that MR(+) supertrees also generalize MR consensus in this case: For bifurcating input trees on the same taxon set, MR(+) cannot place missing taxa or resolve multifurcations and thus MR(+) directly minimizes the RF distance to the input trees. Therefore the distinction between majority-rule consensus and majority-rule(+) consensus is only important for non-bifurcating input trees.

Although these respective consensus methods behave differently in the general case [27], they are equivalent for bifurcating input trees, and I will treat all three methods, MR(-), MR(+)g, and MR(+) as supertree methods generalizing MR consensus for the remainder of the paper.

#### Heuristic algorithm

A heuristic search is necessary to finds upertrees with the minimal score. The three scores and a heuristic to search for supertrees with the minimal score were implemented in the python program PluMiST (Plus and Minus SuperTrees, available from http://www.cibiv.at/software/plumist). The program takes bifurcating trees as input in the case of MR(+) and MR(+)g, and arbitrary trees in the case of MR(-). The algorithm to compute a MR supertree proceeds in the following steps (see methods for details):

1. **Generation of the starting tree **(the starting tree may also be provided by the user).

2. **Supertree **computation by minimizing the respective score functions on bifurcating supertrees. A heuristic tree search using the rearrangement operations TDR (taxa-deletion-reinsertion) and NNI (nearest-neighbor interchange) is carried out.

3. **Strict consensus tree **computation of the best scoring supertrees. The strict consensus contains the splits present in all supertrees.

4. **Contracted consensus tree **computation by deletion of splits that are contradicted by ≥ 50% of the input trees.

The resulting tree, i.e., the contracted consensus tree, is the MR supertree of the respective MR method. The last step contracts splits that violate the MR consensus property. Since the MR consensus tree only contains splits occurring in > 50% of the trees, it cannot contain splits contradicting ≥ 50% of the input trees. Note that this step would be redundant if the tree search was over multifurcating trees. I will also present results without the last step of the algorithm. The respective supertree methods are denoted . That means, the  supertree is the strict consensus tree. The  methods are not generalizations of the majority-rule consensus.

### Testing

Several simulations were conducted to assess the performance of PluMiST. The methods were also compared to MRP using PAUP* [30] with the following options: maximal 1,000,000 trees in memory, 10 replications, and TBR branch swapping.

#### Simulation with compatible input trees

This setting is similar to the setting used in [15]. However, I use different model trees generated under a Yule model [31] and subsequently prune a fraction of taxa randomly from ten input trees. If the pruning step deleted the same taxon from each input tree, the data set was discarded and a new data set was generated instead. Thus each taxon had to be present in at least one input tree. I use the following parameter settings: The number of taxa is 32 or 64, and the fraction of deleted taxa in each input tree is 25% or 50%. 100 replicates are performed for each of the four possible combinations.

An MR method is *successful *if a score of 0 is found and the resulting strict consensus tree contains only splits present in the true tree. Note that this definition of success differs from the one in [15]. In their case, the method is successful only if the true tree was the only supertree. However, I think that a method should not return only one best scoring tree if some nodes cannot be resolved. Multiple trees with a score of 0 are clearly an indication that some nodes cannot be resolved. In the simulations, there was no check for sufficient overlap between the input trees. Different measures for this criterion exist (e.g., [32,33]). If there is not sufficient overlap, then the supertree cannot be expected to be reconstructed without ambiguity and this should be reflected by multiple supertrees.

**Figure 2:**
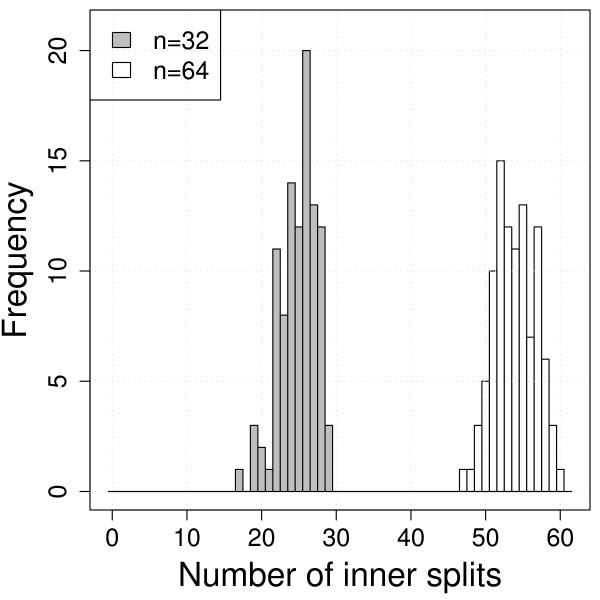
**Distribution of inner splits with compatible input trees and 50% of the taxa deleted**.

With this criterion of success, all three MR methods and MRP were successful in all cases and under all parameter settings. Furthermore, in each simulation, all three MR methods returned the same number of trees. PAUP* may return more trees since it also returns unresolved trees if they have the same parsimony length. However, in all cases, where PAUP* returned more trees, the strict consensus tree (i.e., the supertree) was the same as for PluMiST. With a deletion probability of 0.25, more than one optimal tree is found only three and two times with 32 and 64 taxa, respectively. In these cases the resulting supertree had one split missing. With a deletion probability of 0.5, substantially more optimal trees were found which resulted in more multifurcating trees (Figure 2). On average, trees with *n *= 32 contain 24.9 inner splits (instead of 29 for a bifurcating tree) and with *n *= 64, there are 53.9 inner splits (instead of 61).

#### Simulation with incompatible input trees

**Figure 3:**
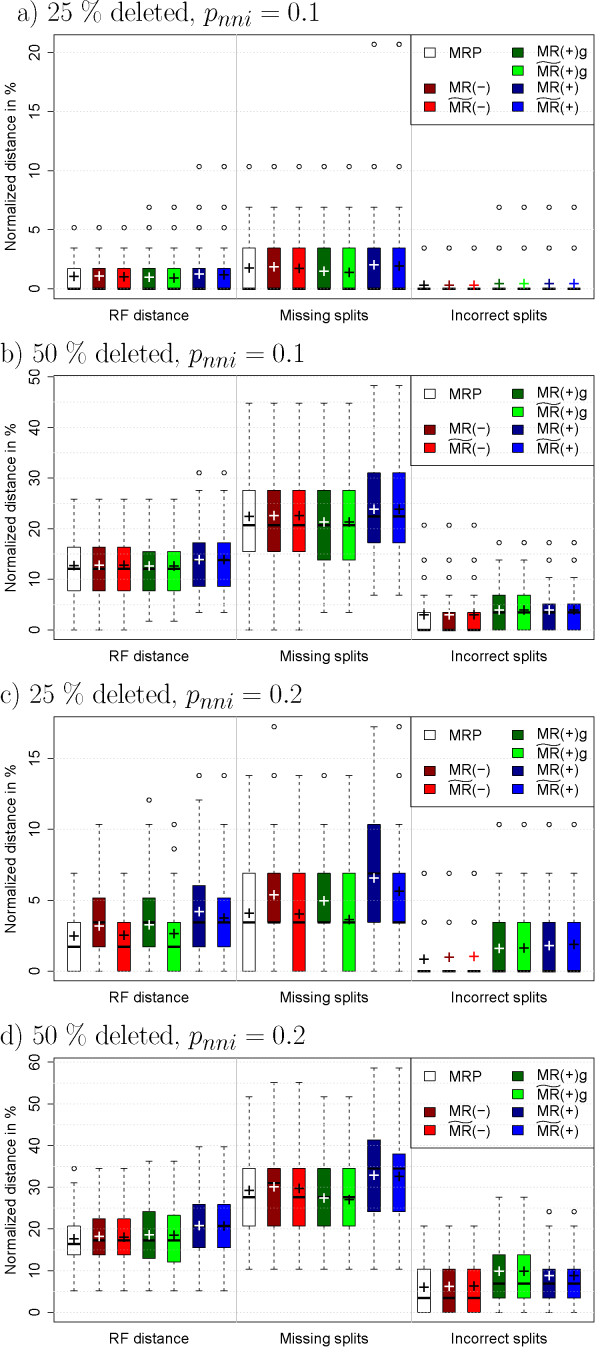
**Results for incompatible input trees and *n *= 32**. The distances are normalized by dividing by the maximal distances, that is, 2*n - *6 for the RF distance and *n - *3 for the other two distances. "Missing splits" is the proportion of splits that are in the model tree but not in the supertree and "Incorrect splits" are in the supertree but not in the model tree. The mean of the data is displayed by "+". Note the different scaling of the *y*-axes.

I use the same model trees and input trees as in the previous section. However, the input trees were modified such that each internal edge undergoes a nearest-neighbor interchange (NNI [34]) with probability *p_nni _*and stays the same with probability 1*-p_nni_*. The two alternative NNIs are equally likely. The results for *n *= 32 and *p_nni _*of 0.1 and 0.2, respectively, are shown in Figure 3.

Since MRP usually performed well in these simulations, its average distance is used as a baseline and I report to what amount the MR methods exceed it. The results for MR(-) are generally comparable to the results for MRP. The difference in the mean distances is less than 0.8% for  and less than 0.5% for MR(-) and all simulations with *n *= 32. In contrast, for the most difficult simulation (Figure 3d), the mean distances for MR(+)g and  exceed the ones for MRP by 1% and 0.8%, respectively, and the mean distances for MR(+) and  exceed the mean distance for MRP by 3.2% each. The methods show differences when only the number of missing splits is considered: The average number of missing splits for MR(-), , MR(+)g, and  increases the average for MRP by less then 0.7% each. In contrast, MR(+) and  miss up to 1.8% more of the true splits.

MR(-) rarely finds more incorrect splits than MRP. MR(-) and  do not find more incorrect splits on average for *p_nni _*= 0.1, and 0.1% more for *p_nni _*= 0.2. In contrast, MR(+)g and  find up to 1.9% more incorrect splits, while MR(+) and  find up to 0.8% more incorrect splits.

#### Sequence simulation

PluMiST was also incorporated into a supertree simulation pipeline [35]. Two simulation settings were carried out: a *small simulation *with 25 taxa, 10 input trees, and on average 37.5% of the taxa deleted and a *large simulation *with 69 taxa, 254 input trees, and on average 84.2% of the taxa deleted. Here I present the results for the simplest setting where the true gene trees are subtrees of the species tree and the simulation parameters are the same for all genes. Input trees were generated by maximum likelihood reconstruction from simulated alignments. 500 simulated data sets were evaluated for the small simulation and 200 for the large simulation.

In contrast to the previous simulations, I conducted ten independent replicates for all MR supertree computations and combined trees with the best score over all runs into the final supertree. However, these results were very similar compared to taking one run only (data not shown). Thus, I conclude that the combined search heuristic of TDR and NNI is a sufficient exploration of the tree space in these simulations and report and discuss the results for one run only.

**Figure 4:**
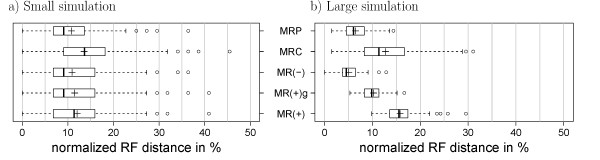
**Results with sequence simulation**. The mean of the data is displayed by "+".

The results for the small simulation (Figure 4a) are similar to the results from the previous section: MR(-) has a slightly higher distance than MRP: 10.9% compared to 10.8%; MR(+)g and MR(+) have higher distances of 11.5% and 12%, respectively. The differences between the MR methods are more pronounced in the large simulation (Figure 4b). Here, MR(-) (average distance of 5.1%) clearly outperforms MR(+)g (10.2%) and MR(+) (15.8%). In both simulations, the -versions of the MR methods resulted in the same trees.

Furthermore, MR(-) clearly improves another MRC implementation (Clann version 3.0.2 [36] with the sfit criterion, SPR search, nsteps = 3, maxswaps = 1000000, 1 repetition). In the large simulation, MR(-) also outperforms MRP (6.5%). Both the percentage of missing splits (7.1% with MR(-) compared to 8.6% with MRP) and of incorrect splits (3% compared to 4.3%) improves.

There are two main reasons why different methods might reconstruct different trees: the scoring function and the search heuristic. To evaluate whether the difference in the scoring functions can explain the distance differences observed for the large simulation (Figure 4b), the trees of all four methods, MRP, MR(-), MR(+)g, and MR(+) were scored with the other objective functions. If a method found multiple trees, all trees were scored and the minimum was taken. In > 90% of the cases the MR(+) supertree had a smaller MR(+) score than any other supertree, and this also holds for MR(+)g. MRP and MR(-) supertrees also usually have a smaller parsimony lengths and MR(-) scores, respectively, than the MR(+)g and MR(+) trees (> 99% of the cases). In 31% of the cases the parsimony lengths of the MRP and MR(-) supertree were equal and in 31.5% the MR(-) scores of both methods were equal. It was never observed that another method found a lower parsimony or MR(-) score than the respective supertree methods. However, for MR(+), at least one method resulted in a better MR(+) score in 6.5% of the cases. In 0.5% of the cases, one MR(+) supertree had a better MR(+)g score than the MR(+)g supertree.

To summarize, supertrees from different methods usually vary in their scores when evaluated with one objective function (MRP, MR(-), MR(+)g, or MR(+)). The search heuristics implemented in PAUP* and PluMiST usually find better scores for the respective objective functions compared to the supertrees found with the other methods.

#### Evaluation of real data sets

The program was also applied to real data sets and compared with MRP using PAUP*. First, two data sets also used in Bansal et al. [29] were analyzed (available from [37]). The seabirds data set [38] contains rooted trees, thus an outgroup taxon was added to the trees. The mammals data set contains "semi-rooted" trees [39], i.e., not all trees are rooted and an outgroup taxon is already present in some of the input trees. Some trees were discarded from the mammal data set since they contain no inner splits and thus no information for MR supertrees or MRP. Since both data sets contain some multifurcating input trees, only MR(-) was evalutated.

The results are summarized in Table 2. The high number of optimal trees for the seabirds data set had already been reported [38]. This slows down the last part of the PluMiST algorithm, because equally scoring trees are explored by NNI. The RF supertree method [29] finds a score of 61, but only 4 trees of the optimal score for this data set. This score also corresponds to the optimal MR(-) score of 23, but PluMiST finds 1538 optimal trees. The runtime of RF supertrees is substantially lower than the runtime of PluMiST or PAUP* for two reasons. First, efficient heuristics use the root information, and second, the search does not continue to find multiple optimal trees.

**Table 2:** Results with the seabird and the mammal data set

MR(-)						
Data Set	Taxa	Input trees	Best score	Optimal trees	MRP-score	Time
Seabirds [38]	122	7	23	1538	214	1 day*
Mammals [39]	116	692	2160	272	9454	13h10

						
**MRP**						
**Data Set **	**Taxa**	**Input trees**	**Best score**	**Optimal trees**	**MR(-)-score**	**Time**

Seabirds [38]	122	7	214	10^6^	23	9h00
Mammals [39]	116	692	9452	109	2162	1h40

* The search with the seabirds data set was aborted after about 1 day, and the optimal trees saved thus far were used. The optimal score of 23 was found after 1h20.

**Figure 5:**
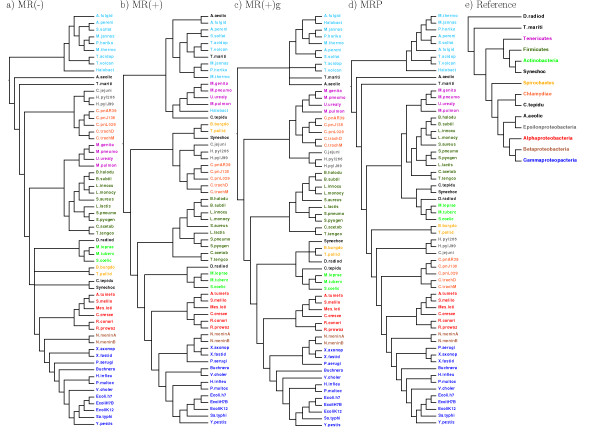
**Prokaryote trees**. Reference tree for the bacteria is taken from Wu et al. [41]. *Chlorobium tepidum *is not present in [41] but is displayed at the position of the other *Chlorobi*. The Archaea are marked in turquoise. Full taxon names can be found in [40].

The mammal data set has different optima depending on whether the MRP or the MR(-) criterion is used (Table 2). The full data set could not be analyzed with RF supertrees since not all trees are rooted. Lastly, a microbial data set containing 61 taxa and 1117 genes was analyzed [40]. Bootstrap resampling of the gene trees and computing MRC using Clann had resulted in a highly multifurcating majority-rule consensus tree [40]. The data set consists of bifurcating unrooted trees and I applied it to the three majority-rule supertree methods and to MRP. All supertrees are completely or nearly completely resolved (Figure 5). There are clear differences in the scores of the optimal trees when evaluated with other scoring functions (Table 3). None of the trees matches a recent reference phylogeny completely (Figure 5e, Figure 1 in [41]). All of the nine groupings marked by different colors in Figure 5 are present in the MR(-) and in the MRP tree. Both of the MR(+) variants nest the *Betaproteobacteria *inside the *Gammaproteobacteria*. The branching order of these groupings also shows some deviations from the reference tree. First, most of the groupings with only one member in the data set are not correctly placed in any tree: *Aquifex aeolicus *is never a sister of the *Epsilonproteobacteria*, *Chlorobium tepidum *is never a sister of the *Chlamydiae*, the *Cyanobacterium Synechocystis *is never a sister of the *Actinobacteria*, and *Deinococcus radiodurans *is never basal. Because of these problems, the information about groupings with one member only are ignored in the following points. Second, the *Alpha-*, *Beta- *and *Gammaproteobacteria *form a clade in all trees, but the *Epsilonproteobacteria *are not their sister group. In the MR(-) and MR(+) tree, the *Epsilonproteobacteria *form a clade with the *Chlamydiae *and in the MR(+)g tree, they form a clade with the *Chlamydiae *and the *Tenericutes*. In the MRP tree, they are basal to a clade of *Chlamydiae *and the other *Proteobacteria*. Third, in both the MR(-) and the MRP tree, the *Firmicutes *do not cluster with the *Actinobacteria *but with the *Tenericutes*. Only in the MRP tree, the clade of *Tenericutes*, *Firmicutes *and *Actinobacteria *is basel to the other *Bacteria*. In the MR(-) tree, the clade of *Epsilonproteobacteria *and *Chlamydiae *is basal and in the MR(+)g tree a clade of these two and the *Tenericutes *is basal. The MR(+) tree has more differences; even the *Archaea *are not monophyletic.

**Table 3:** Results with the microbial data set

	Scores					
			
Method	MR(-)	MR(+)	MR(+)g	Parsimony	Optimal trees	Time
MR(-)	**1453**	1875	3328	6802	4	19 min
MR(+)	1539	**1744**	3283	7003	1	24 min
MR(+)g	1484	1753	**3243**	6862	4	36 min
MRP	1474	2010	3484	**6775**	1	2 sec

Scores matching the criterion used for optimization are marked in bold.

Taken together there are serious problems in the data set, when groups have only one member. The fewest contradictions to the reference tree are present in the MRP tree followed by the MR(-) tree. Note that the deep branching of the bacterial phylogeny and the meaningfulness of a search for a tree like pattern in *Bacteria *is hotly debated (e.g., [42-45]). Here, the microbial data set is presented as an example how different methods resolve conflict among the input trees and not as a statement about the "true" bacterial phylogeny.

## Conclusions

I present a new framework for the computation of the distances underlying majority-rule supertrees. The basis of this framework is the relationship matrix that stores the possible relationships between an input tree split and a supertree split: subsplit, compatibility, or incompatibility. The distance computations for MR(-), MR(+)g, and MR(+) are presented for bifurcating input- and supertrees. The distance computation of MR(-) also applies to multifurcating input trees.

These distance computations are implemented in the python program PluMiST. The program was tested in a simulation study with different settings, in particular with compatible and incompatible input trees. With compatible input trees, all MR methods find a score of 0 and the same supertree as MRP and I thus conclude that all methods search the tree space successfully. With incompatible input trees, the results for MR(-) are best, especially when both missing and incorrect splits are taken into account. MR(+)g might miss less splits than MR(-), however it also finds more incorrect splits. Reconstructing incorrect splits which give rise to false conclusions is obviously a more serious problem compared to the exclusion of some splits.

The RF distance to the true tree is often decreased by including splits contradicted by a majority of the input trees (i.e., skipping the last step of the algorithm, denoted by ). This holds particularly when only a few taxa are deleted and the input trees are highly incongruent (25% of the taxa deleted and *p_nni _*= 0.2). While the  methods are not a generalization of MR consensus anymore, they may still be useful supertree methods. Given that  is equivalent to MRC for bifurcating supertrees, PluMiST can then also be used as a heuristic for MRC.

In a more realistic simulation setting involving more input trees, trees of different sizes and different amounts of missing data and sequence simulation, MR(-) performed very well. It outperformed the MRC implementation in Clann and also MRP for the large data set. Those differences can be traced back to the differences in the objective functions in PAUP* and PluMiST. The trees found with the respective objective function usually have better scores compared to the supertrees found with other methods.

This is also observed when analyzing biological data sets. Of the three data sets analyzed, two contain multifurcating input trees, and therefore only MR(-) was compared to MRP and to RF supertrees. For one data set with rooted input trees the score of MR(-) was equivalent to the score to RF supertrees, but MR(-) finds more optimal trees. The microbial data set contains bifurcating trees and was analyzed with all three majority-rule supertree methods. The MR(-) tree recovered more relationships present in the reference tree compared to the MR(+) variants.

In conclusion, the objective function of MR(-) performs best among the MR supertree methods studied here. Note that this objective function is also justified in the likelihood setting [46]. The MR(+) supertree methods add taxa to the input trees and apply a consensus. At first view, this approach seems to be a natural method for dealing with the supertree problem. However, phylogenetic signal may be confounded by properties of the complex tree space [47].

The problem of distance computations in the general case of multifurcating trees is still open but I suppose that formulas for these cases can be constructed based on the relationship matrix. The generalization of these computations to multifurcating supertrees is an important task. The proof for majority-rule consensus trees [19] only holds for multifurcating consensus trees and was the motivation for majority-rule supertrees [26]. Note that another variant of MR(+), MR(+)s supertrees, can be computed via the span and the consensus step and thus also includes multifurcating trees [28].

The current implementation of MR(-) in PluMiST is similar to the RF supertree method. The approach of Bansal et al. [29] is, however, to find at least one tree of the optimal score and not to search for equal scoring trees. This latter property is needed for MR supertrees. This, in addition to the heuristics for rooted trees, makes the RF supertree method fast. In contrast, the advantage of MR(-) is that input trees can be unrooted and that the area around the optimum is searched for equally scoring trees.

Furthermore, PluMiST and the theory presented here comprises a general framework for MR supertrees. The relationships between the distance functions underlying these methods will hopefully help in understanding the similarities and differences of the methods. Simulations show that MR(-) usually performs very well, in particular in comparison with the established MRP method and is thus recommended for majority-rule supertree reconstruction.

## Methods

### Phylogenetic Background

A (phylogenetic) tree is a leaf-labeled tree and is thus identified by its leaf set *X *and its edge set (for details and terminology see also [48]). The leaves are usually called taxa. *Terminal *edges connect a leaf with an inner node and *inner *edges connect two inner nodes. I present the computation for *unrooted *trees. This computation can easily be applied to rooted trees by treating the root as an additional taxon. In unrooted trees there is no node of degree two.

If an edge of a phylogenetic tree is deleted, the tree decomposes into two connected components. Thus, the taxon set is then partitioned into two sets (*X*_1 _and *X*_2_), one for each component. Such a bipartition is called a *split *and is denoted by *X*_1_|*X*_2_. Since each edge in a tree corresponds to a split, a tree on taxon set *X *is identified by the corresponding split set (see example in Figure 1, note that the taxon sets in a split can be shortly written as a string of concatenated taxa).

Two splits are called *compatible *if there is a phylogenetic tree containing both splits. This holds for two splits *X*_1_|*X*_2 _and *Y*_1_|*Y*_2 _if at least one of the following taxon sets is empty: *X*_1 _∩ *Y*_1_, *X*_1 _*∩ Y*_2_, *X*_2 _*∩ Y*_1 _or *X*_2 _*∩ Y*_2_. Note that terminal splits are compatible to any other split. An unrooted phylogenetic tree of *n *taxa contains at most *n - *3 inner splits. If it contains exactly *n - *3 inner splits, all inner nodes have degree three, and the tree is called *bifurcating*, or *multifurcating *otherwise. An inner node of degree three is a taxon tripartition and can be written as *X*_1_|*X*_2_|*X*_3_.

A tree *T*_1 _*displays *a tree *T*_2 _if it contains all splits of *T*_2_, i.e., *T*_2 _⊆ *T*_1_. The *Robinson-Foulds *(RF) distance of two trees is defined as the symmetric difference of the split sets [20]: RF(*T*_1_, *T*_2_) = | *T*_1_\*T*_2 _| + | *T*_2_\*T*_1_|. Note that if both trees are bifurcating, then both set sizes are equal and RF is an even number.

A supertree  for a set of input trees  is a tree that contains exactly the taxa occurring in at least one input tree, i.e. . Thus in general . The following abbreviations are used:  and . The splits in  are called *partial *if . In contrast, the splits in  are called *plenary*. A partial split  is a *subsplit *of a plenary split  if one of the following conditions holds: (*Y*_1 _⊆ *Z*_1 _and *Y*_2 _⊆ *Z*_2_) or (*Y*_1 _⊆ *Z*_2 _and *Y*_2 _⊆ *Z*_1_). For example, the split *ABC*|*F *is a subsplit of the split *ABC*|*DEF*. Two splits on different taxon sets will be called compatible if they are *compatible *on the set of taxa occurring in both trees and *incompatible *otherwise.

 is the *restriction *of tree  to taxon set , i.e., .

### Consensus methods

Consensus methods combine input trees on the same taxon set. There are many consensus methods available [17], some of which are based on the split sets of the input trees:

**Strict consensus **The strict consensus contains all splits present in all input trees, i.e., .

**Majority-rule consensus **The majority-rule (MR) consensus contains all splits present in more than half of the input trees.

### Distance computations for MR supertrees

The majority-rule supertree methods were defined to minimize a particular score [26,27]:

**MR(-) **Find a tree  that minimizes  where .

**MR(+)g **Find a tree  that minimizes  where  and 

**MR(+) **Find a tree  that minimizes  where 

and 

**Figure 6:**
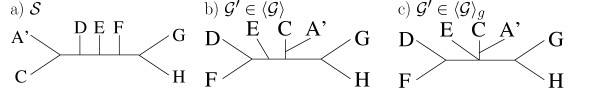
**Modification of the supertree**. The taxon set {*A, B*} is replaced by *A*'.

As for majority-rule consensus trees, the majority-rule supertree is the strict consensus of all potentially multifurcating trees that minimize the distance. Here, the tree search is restricted to bifurcating trees. In the following I show, that for bifurcating input- and supertrees the distance computations can be simplified. Therefore assume that  is already modified as follows: If there is an inner split  with  and there is no split  with , then replace the subtree spanned by the the taxa in *X_i _*by a dummy taxon (*i, j *∈ {1, 2}). For *d^- ^*this modification will make no difference, since the dummy taxon is deleted again. For *d*^+ ^and  only this dummy taxon needs to be placed in . Afterwards it could be expanded again without any cost. This modification ensures that in the optimal  each taxon was added to an edge already existing in  (analogous for ). The modified example tree is shown in Figure 6.

A *matrix representation *is a binary coding of the splits, an example is shown in Table 4. Furthermore, the *relationship matrix *of dimension  is used, which indicates for each pair of input-and supertree splits, whether they are subsplit; compatible but no subsplit; or incompatible. Only inner splits and trivial splits for  are included (see Table 4). Note that for bifurcating trees there cannot be a split compatible to all other splits in the relationship matrix. Thus each line and row, respectively, must contain either at least one *s *or at least one *i*.

**Table 4:** Coding of modified example trees

**Matrix representation**																	**Relationship matrix**										
													
													
	***s***_**1**_	***s***_**2**_	***s***_**3**_	***s***_**4**_	***s***_**5**_	***s***_**6**_	***s***_**7**_	***s***_**8**_	***s***_**9**_	***g***_**1**_	***g***_**2**_	***g***_**3**_	***g***_**4**_	***g***_**5**_	***g***_**6**_	***g***_**7**_		***s***_**1**_	***s***_**2**_	***s***_**3**_	***s***_**4**_	***s***_**5**_	***s***_**6**_	***s***_**7**_	***s***_**8**_	***s***_**9**_	***c***_***i***_
	
A'	1	1	0	0	0	0	0	0	0	-	-	-	-	-	-	-	*g*_1_	c	i	i	c	c	c	c	c	c	1
C	1	1	0	0	1	0	0	0	0	0	0	1	0	0	0	0	*g*_2_	c	c	c	s	c	c	c	c	c	0
D	0	1	0	0	0	1	0	0	0	1	0	0	1	0	0	0	*g*_3_	s	c	c	c	s	c	c	c	c	0
E	0	0	0	0	0	0	0	0	0	-	-	-	-	-	-	-	*g*_4_	c	c	c	c	c	s	c	c	c	0
F	0	0	1	0	0	0	0	0	0	1	0	0	0	1	0	0	*g*_5_	c	c	c	c	c	c	s	c	c	0
G	0	0	1	1	0	0	0	0	0	0	1	0	0	0	1	0	*g*_6_	c	c	c	c	c	c	c	s	c	0
H	0	0	1	1	0	0	0	0	1	0	1	0	0	0	0	1	*g*_7_	c	c	c	c	c	c	c	c	s	0
	
																	*b_j_*	0	1	1	0	0	0	0	0	0	

MR(-)

**Theorem 1 **Given a bifurcating supertree  and a bifurcating input tree . Then *d^- ^*= 2*C*, where *C *is the number of splits in  that are incompatible to at least one split in .

**Proof **Let  be the restriction of  to , i. e. .

. Since both trees are bifurcating, each  must be either identical or incompatible to a split in . Thus  measures the number of splits in  that are incompatible to a split in . Each  that is incompatible to a split in , is also incompatible to a split in , since incompatibility can only be caused by the taxa in . Thus  is the number of splits in  that are incompatible to a split in .   ■

**Note **The formula easily generalizes to multifurcating input trees: Assume that  has *m *multifurcations, i.e.,  inner branches. Then *d^- ^*= 2*C *+ *m*, there are *C *splits in  that conflict with , *C *splits in  that conflict with , and in addition *m *splits in  that are missing in . Since *m *is constant for all supertrees, the objective function is equivalent to the one for bifurcating input trees.

Note, that *C *can be easily computed from the relationship matrix since .

MR(+)g

**Theorem 2 **Given a bifurcating supertree  and a bifurcating input tree . Then , where *B *is the number of splits in  that are incompatible to at least one split in  and *C *is the number of splits in  that are incompatible to at least one split in .

**Proof **Both inequalities are shown. The idea of the proof is to show that  and  for one . Since *B *≥ *C*, , this is accomplished by potentially introducing multifurcations when placing taxa onto .

 For all  and . Thus this inequality holds for all .

 Need to construct a  with .

First, . It is shown now that the taxa in  can be placed onto  and  and thus increase the distance by not more than *B - C*. Therefore, . *n_T _*= *n - n_G _*taxa have to be placed onto  to get . There will be *n_good _*"good" taxa that are placed onto edges without introducing an increase in the distance; and *n_bad _*"bad" taxa that are placed onto nodes and will increase the distance by one; *n_T _*= *n_good _*+ *n_bad_*.

Each supertree split can only be a supersplit of at most one input split. If it is a supersplit of an input split, it is compatible to all others. An input split may be a subsplit of different supertree splits. If it is a subsplit of > 1 supertree split, each additional supertree split gives information about the placement of one taxon. e.g., the input split *A*|*B *may be a subsplit of the supertree splits *AX*|*B *and *A*| *XB *(*A *and *B *are taxon sets, *X *is a taxon). Then *X *is a "good" taxon and placed on the edge between *A *and *B *without increasing . The number of taxa that are placed with this procedure is .  and  represent the respective number of columns and rows in the relationship matrix that have a subsplit entry.

In each row and column of the matrix, there must be either at least one subsplit entry (s) or at least one incompatibility entry (i). Thus  and . Using this, the remaining number of taxa to insert is

These *B - C *bad taxa can be placed on a node increasing  by one. In detail, the two splits in *S *that are adjacent to the terminal split of the bad taxon will be incompatible with at least one split in . As a result the bad taxon can be placed on any of the nodes adjacent to a conflicting split in . The resulting distance is not larger than 2*C *+ (*B - C*) = *B *+ *C*.    ■

**Example **In the example , , *C *= 1 and *B *= 2. Thus,  (taxon *A*') and *n_bad _*= *B - C *= 1 (taxon *E*). *g*_3 _is a subsplit of both *s*_1 _and *s*_5_, thus *A*' can be placed on the terminal edge leading to *C *without conflict. *s*_2 _and *s*_3 _are adjacent to the terminal split of *E*. They are conflicting with *g*_1 _and thus *E *can be placed either on the node *D*|*F*|*CGH *or on the node *C*|*DF *|*GH*.

MR(+)

**Theorem 3 **Given a bifurcating supertree  and a bifurcating input tree . Then *d*^+ ^= 2*B*, where *B *is the number of splits in  that are incompatible to at least one split in .

**Proof **The proof is a modification of the proof for Theorem 2. The "good" taxa are placed in the same way and the bad taxa are placed *onto *any split corresponding to a conflicting split in . The resulting distance is then 2*C *+ 2(*B - C*) = 2*B*.    ■

### Heuristic algorithm

#### Starting tree

The tree search starts with a *step-wise addition *tree. A random taxa order is processed the following way: The quartet toplogy for the first four taxa is determined by the topolgy most frequent among the input trees. The remaining *n - *4 taxa are inserted step by step to the partially reconstructed tree. For each of the remaining taxa, the informative input trees are determined. These trees contain the considered taxon and at least 3 of the taxa already inserted. Afterwards, the best insertion point of the terminal branch labeled with the taxon is determined: If the objective function is *d^-^*, then the sum of *d^- ^*is computed for each insertion point. This is done by pruning the supertrees and the input trees to the common taxon sets. If the objective function is *d*^+^, for each split in the supertree the number of input trees it contradicts is determined. The insertion point minimizes the sum of the split contradictions. This method does not ensure *d*^+ ^since taxa are missing in both trees. If the objective function is , both types of insertion strategies are carried out alternatingly, i.e., for each insertion only one of the strategies is used. In all cases ties are resolved randomly.

#### Optimization step

The tree search allows for two rearrangement operations: Taxa-deletion-reinsertion (TDR) and nearest-neighbor interchange (NNI). Given a tree, TDR deletes a given fraction of the taxa randomly (by default 0.25). Afterwards, a random taxon order of the deleted taxa is determined and the taxa are reinserted via the step-wise addition strategy. An NNI operation takes a split of a tree and generates the two alternative splits that could replace it. If replacing the split with one of these splits would result in a supertree with a lower score, the split is replaced. Alternative best trees are also returned.

The tree search proceeds in two stages: The first *exploration *stage lasts at most *l*^2 ^iterations, where *l *is the number of inner splits in a supertree and an iteration is an NNI or a TDR operation. First, all splits of the starting tree are optimized by NNI until there is no further improvement. Next, the tree space is explored by TDR. After one TDR operation, all splits of the new tree are optimized by NNI again. It may happen that a TDR operation generates a tree that it found before or that yield no improvement with NNI. Then this tree is discarded and a new tree is generated with TDR. If  trees were discarded consecutively or *l*^2 ^iterations passed, the *broadening *stage starts. At this point, all optimal trees that have not been analyzed before, are optimized by NNI. This mainly ensures that all trees in the optimal region are found.

In the end, all optimal trees are summarized by a strict consensus and splits that are contradicted by ≥ 50% of the input trees are deleted. In these trees, the internal nodes are labeled with support values similar to those of majority-rule consensus trees. Each inner node is labeled with *x/y*, where *x *is the number of input trees not contradicting the corresponding split and *y *is the number of input trees where a nontrivial split supports the corresponding split. Thus, *y *is the number of input trees that *support *the node and *x - y *is the number of input trees that are *irrelevant *for that node with the definitions of support and irrelevance used in [49].

## Authors' contributions

AK conceived and implemented the method, carried out the analyses, and wrote the manuscript.
